# Impact of institutional treatment guidance on the management and outcomes of *Stenotrophomonas maltophilia* and carbapenem-resistant *Acinetobacter baumannii* infections

**DOI:** 10.1017/ash.2025.10244

**Published:** 2025-12-10

**Authors:** Ryan Vathy, Christo Cimino, Ben Ereshefsky, Romney Humphries

**Affiliations:** 1 Department of Pharmaceutical Services, Vanderbilt University Medical Center, Nashville, TN, USA; 2 Department of Pathology, Microbiology, and Immunology, Vanderbilt University Medical Center, Nashville, TN, USA

## Abstract

**Objective:**

To evaluate the impact of a guidance document on treatment decisions and clinical outcomes for infections caused by carbapenem-resistant *Acinetobacter baumannii* (CRAB) and *Stenotrophomonas maltophilia.*

**Design:**

This was a single-center, retrospective, quasi-experimental, pre- and post-intervention study. The primary outcome was adherence to antibiotic selection and dosing as recommended by the guidance document. Secondary outcomes included clinical efficacy, readmission within 90 days, all-cause 30-day mortality, 90-day microbiologic recurrence, and antibiotic-associated adverse effects.

**Setting:**

The study was conducted at a large, academic medical center in the Southeastern United States.

**Participants:**

The study included adult patients who were admitted to our institution from November 2018 to June 2024 who grew CRAB or *S. maltophilia* from any culture and were definitively treated.

**Intervention:**

This study included an institution specific treatment algorithm for the treatment of infections caused by resistant gram-negative organisms published on June 1^st^, 2022, on our institution’s antimicrobial stewardship website.

**Results:**

Twenty-two patients with CRAB (14 pre- and 8 post-intervention) and 150 with *S. maltophilia* (75 pre- and 75 post-intervention) were evaluated. For CRAB, 0 (0%) patients in the pre-intervention group and 5 (62.5%) in the post-intervention group met the primary outcome (*P* < .001). For *S. maltophilia,* 21 (28%) patients in the pre-intervention group compared to 38 (50.7%) in the post-intervention group met the primary outcome (*P* < .004). Secondary outcomes did not differ significantly pre- and post-intervention.

**Conclusions:**

These findings demonstrate that implementing a local treatment guideline based on IDSA guidance may increase the utilization of evidence-based directed antimicrobial therapy for CRAB and *S. maltophilia.*

## Key points

After the implementation of an institutional guideline was shared with infectious diseases providers, we saw a significant change in treatment regimens for infections caused by carbapenem-resistant *Acinetobacter baumannii* and *Stenotrophomonas maltophilia.*

## Background

Antimicrobial resistance is a significant threat to public health due to the prevalence of multidrug-resistant (MDR) bacterial infections with an estimate of more than 2.8 million infections and 35,000 deaths occurring annually.^1^ MDR bacteria are defined as isolates with non-susceptibility to at least one agent in three or more antimicrobial classes, and extensively drug-resistant (XDR) bacteria are defined as isolates non-susceptible to at least one agent in all but two or fewer antimicrobial classes.^2^ Infections caused by MDR and XDR gram-negative bacteria are associated with increased morbidity and mortality.^3^ Therefore, ensuring appropriate antimicrobial therapy is started for patients with these infections is essential.^3^ Because of the significant threat to public health and a high morbidity and mortality associated with these infections, Infectious Diseases Society of America (IDSA) convened a panel of experts who annually publish guidance on the treatment of MDR gram-negative infections.^4^ In December 2021, the first version of this guidance document was published and addressed the treatment of carbapenem-resistant *Acinetobacter baumannii* (CRAB) and *Stenotrophomonas maltophilia* infections.^4^ Based on the guidance document published by IDSA, an institution specific treatment algorithm for the treatment of infections caused by resistant gram-negative organisms was published on June 1^st^, 2022, on our institution’s antimicrobial stewardship website. Education about this document was also provided to the Infectious Diseases (ID) Division.

The institutional treatment algorithm was consistent with IDSA’s guidance as well as our local susceptibility patterns. This document included recommended empiric agents, when to use combination therapy, and appropriate dosing. Notable treatment options included high-dose ampicillin-sulbactam (9g IV q8h over 4h) as a preferred agent for CRAB either alone or in combination with other active agents for moderate to severe infections.^4^ For *S. maltophilia,* this document recommended sulfamethoxazole-trimethoprim (SXT), high-dose minocycline (200 mg IV/PO q12h), tigecycline, levofloxacin, or cefiderocol monotherapy for mild infections with SXT being the preferred agent.^4^ For moderate to severe infections, combination therapy with any of the previously mentioned agents was recommend or the combination of ceftazidime-avibactam and aztreonam as an alternative if intolerance or inactivity to other agents previously mentioned.^4^ While this document provided helpful guidance for clinicians, it is important to note that there is no standard of care regimen for either organism, and it is difficult to differentiate colonization from infection when these organisms are recovered in culture, especially for critically ill patients.^4,5^

The impetus for implementation of an institutional guideline was to improve patient care as well as increase compliance with The Joint Commission’s requirement of adopting evidence-based guidelines to improve antimicrobial use.^6^ The purpose of this study was to evaluate treatment strategies and measure adherence to this guidance document at our large academic medical center.

## Methods

This was a single-center, retrospective, quasi-experimental, pre- and post-intervention study of adult patients who were admitted to Vanderbilt University Hospital from November 1^st^, 2018, to June 1^st^, 2024, who grew CRAB or *S. maltophilia* from any culture and were treated with antibiotics targeted against these organisms. Patients were identified by electronic medical record search after a list of isolates was pulled by our microbiology lab during the study time period. The study was approved and deemed exempt from consent by the VUMC IRB (IRB ID: 241736).

On June 1^st^, 2022, this treatment algorithm was published on the ID clinical documents webpage for all providers to access (Supplementary Figure 1). Education on the institution-specific algorithm was provided to the Vanderbilt University Medical Center (VUMC) ID Division during the weekly noon conference. While there is no institution policy or microbiology nudge recommending an ID consult for these organisms, this treatment algorithm was created by ID/antimicrobial stewardship pharmacists who were routinely reviewing patients with positive cultures with CRAB or *S. maltophilia*. ID pharmacists also provided education during ID rounds to help increase uptake and utilization of the document.

Our microbiology laboratory initially identified 74 isolates for *A. baumannii* and 869 isolates of *S. maltophilia* within the specified pre- and post-intervention periods of November 1^st^, 2018–October 31^st^, 2021, and July 1^st^, 2022–June 30^th^, 2024, respectively. Patients were then excluded if they died within 24 h of index isolate collection, did not receive targeted treatment for the organism, <18 years of age, were not carba-R for *A. baumannii*, or not admitted to our institution at the time of treatment. All CRAB isolates that met inclusion criteria were included. For *S. maltophilia* isolates, due to the large sample size and need for manual chart review for many of the secondary outcomes, we chose to include 150 patients, 75 in the pre-intervention group and 75 in the post-intervention group. The 150 *S. maltophilia* patients were selected after the list of isolates was randomized to include the first 75 patients who met inclusion criteria in each time period. Data for this study was collected and managed using REDCap electronic data capture tools.^7,8^

The primary outcome of this study was adherence to and use of a treatment regimen based on our institutional treatment guideline pre- and post-guidance implementation. As formal institutional guidance would not yet have been available, treatment courses pre-implementation were evaluated on whether they would have been adherent to the guideline. Secondary outcomes included clinical efficacy, which was defined as 30-day microbiologic eradication, survival, and no change in therapy. If the organism regrew within 30 days but was not retreated or therapy was not changed, we counted this as meeting clinical efficacy to control for confounding because both organisms are likely to cause colonization. Other secondary outcomes included hospital readmission within 90 days, 30-day all-cause mortality, 90-day microbiologic recurrence, and antimicrobial-related adverse effects.

For statistical analysis, categorial variables were described using frequencies and percentages. Continuous variables were described using medians and interquartile ranges. Unadjusted comparisons were conducted using Fisher’s Exact tests, Pearson’s χ^2^ tests, and Mann-Whitney tests. Statistical analyses were run utilizing SPSS Version: 29.0.0.0 (241).

## Results

**Figure 1.**
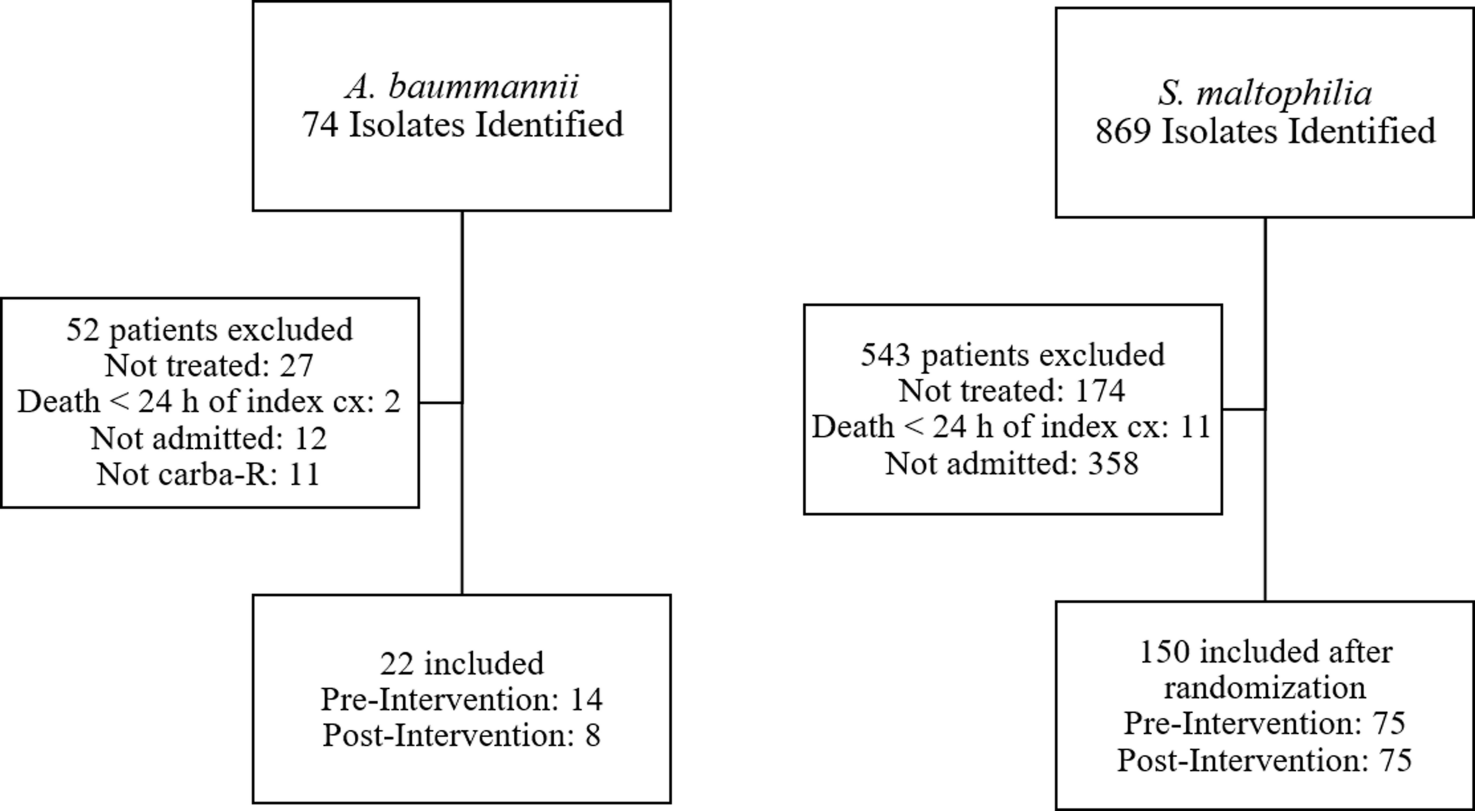
Flowchart of CRAB and *S. maltophilia* patients included in the study.

Twenty-two patients with CRAB (14 pre- and 8 post-intervention) and 150 with *S. maltophilia* (75 pre- and 75 post-intervention) were identified and met inclusion criteria. Reasons for exclusion are shown in Figure 1. Baseline characteristics for the CRAB patients in each group were not found to be significantly different. For *S. maltophilia*, baseline characteristics that significantly differed were age, ICU admission, tracheitis, and co-infection with another organism (Table 1). For the primary outcome in both groups, there was a significant increase in utilization of a guidance concordant treatment regimen based on our institutional document. There were 0 (0%) patients in the pre-intervention group who met the primary outcome and 5 (62.5%) in the post-intervention group (*P* < .001) for patients infected by CRAB. For *S. maltophilia*, 21 (28%) patients in the pre-intervention group met the primary outcome compared to 38 (50.7%) in the post-intervention group (*P* < .004) (Table 2).

**Table 1.** Baseline characteristics

	Pre-intervention CRAB (*N* = 14)	Post-intervention CRAB (*N* = 8)	*P*-value	Pre-intervention *S. maltophilia* (*N* = 75)	Post-intervention *S. maltophilia* (*N* = 75)	*P*-value
**Sex**						
Male *n* (%)	13 (93)	5 (62.5)	0.076	48 (64)	43 (57)	0.40
Female *n* (%)	1 (7)	3 (37.5)	0.076	27 (36)	32 (43)	0.40
Age median, (IQR)	62 (41–65)	46 (39–59)	0.44	53 (41–64)	60 (49–67)	0.007*
BMI median, (IQR)	28.9 (23.9–34.7)	27.1 (25–32.4)	0.97	27.6 (23.4–33.9)	28.4 (22.9–33)	0.98
Immunocompromised *n* (%)	2 (14)	1 (12.5)	0.91	25 (33)	25 (33)	1
ICU admission *n* (%)	9 (64)	5 (62.5)	0.93	44 (59)	60 (80)	0.005*
Trach dependent *n* (%)	5 (36)	0 (0)	0.054	20 (27)	28 (37)	0.16
Vasopressors required *n* (%)	7 (50)	4 (50)	1	28 (37)	35 (47)	0.25
Mechanical ventilation required *n* (%)	8 (57)	3 (37.5)	0.38	33 (44)	44 (59)	0.072
Length of hospital stay, days median (IQR)	25.5 (14–36)	21.5 (14–39.5)	0.95	23 (7–34)	21 (11–45)	0.12
**Type of infection**						
Wound/SSTI *n* (%)	2 (14)	1 (12.5)	0.91	17 (23)	9 (12)	0.08
Pneumonia *n* (%)	2 (14)	2 (25)	1	24 (32)	31 (41)	0.24
Tracheitis *n* (%)	3 (21)	0 (0)	0.16	4 (5)	14 (9)	0.012*
UTI *n* (%)	2 (14)	1 (12.5)	0.91	9 (12)	4 (5)	0.15
Osteomyelitis *n* (%)	3 (21)	2 (25)	0.85	7 (9)	6 (8)	0.77
Intra-abdominal *n* (%)	--	--	--	1 (1)	1 (1)	1
CNS *n* (%)	--	--	--	1 (1)	0 (0)	0.32
Bacteremia *n* (%)	2 (14)	1 (12.5)	0.91	6 (8)	10 (13)	0.29
Endocarditis *n* (%)	--	--	--	2 (3)	0 (0)	0.15
Mediastinitis *n* (%)	--	--	--	1 (1)	0 (0)	0.32
Hardware infection *n* (%)	0 (0)	1 (12.5)	0.44	1 (1)	0 (0)	0.32
Vascular graft infection *n* (%)	--	--	--	1 (1)	0 (0)	0.32
Donor-derived infection *n* (%)	--	--	--	1 (1)	0 (0)	0.32
Treatment Duration, *d* (IQR)	8.5 (7–21)	14 (10.5–43.5)	0.06	14 (7–14)	11 (7–14)	0.25

*P < .05.

IQR, Interquartile Range; BMI, Body Mass Index; ICU, Intensive Care Unit; SSTI, Skin and Soft Tissue Infection; CNS, Central Nervous System.

**Table 2.** Primary outcome

	Pre-intervention CRAB (*N* = 14)	Post-intervention CRAB (*N* = 8)	*P*-value	Pre-intervention *S. maltophilia* (*N* = 75)	Post-intervention *S. maltophilia* (*N* = 75)	*P*-value
Guideline concordant treatment regimen *n* (%)	0 (0)	5 (62.5)	<0.001*	21 (28)	38 (50.7)	0.004*
**Reason for non-adherence to guidance document**						
Incorrect drug dose	9 (64)	2 (25)	0.76	8 (10.7)	9 (12)	0.80
Drug used not on treatment algorithm	6 (43)	1 (12.5)	0.141	11 (14.7)	1 (6.7)	0.003*
Monotherapy used	--	--	--	38 (50.7)	31 (41)	0.25

*P < .05.

In the CRAB patients, the most common reason for non-adherence was the use of incorrect dosing of ampicillin-sulbactam 3g q4-q6h even when accounting for renal dose adjustments or minocycline 100 mg q12h with 9 (64%) in the pre-intervention group, and 2 (25%) patients in the post-intervention group (*P* < .76). In the *S. maltophilia* patients, the most common reason for non-adherence was the use of monotherapy in moderate-to-severe infections. There was also a statistically significant difference in patients in the pre-intervention group being treated with an antibiotic not recommended in the guidance document with 11 (14.7%) versus 1 (6.7%) in the post-intervention group (*P* < .003) (Table 2).

For the secondary outcomes of all-cause 30-day mortality, hospital readmission within 90 days, clinical efficacy, recurrence of an organism within 90 days, and antimicrobial related adverse effects, there was no significant difference between either groups for patients with CRAB or *S. maltophilia* (Table 3). For CRAB, there were several antimicrobials used in the pre-intervention group that were not used in the post-intervention group which included colistimethate, levofloxacin, and amikacin, but these differences were not statistically significant. In the *S. maltophilia* cohort, there were multiple statistically significant differences in the use of antimicrobials. There were more patients in the pre-intervention group treated with ceftazidime with 8 (10.7%) in the pre-intervention group and 1 (1.3%) in the post-intervention group (*P* < .016). There were 49 (65%) patients in the pre-intervention group treated with levofloxacin as opposed to 26 (34.7%) in the post-intervention group (*P* < .001), and 26 (34.7%) patients in the pre-intervention group treated with SXT compared to 41 (54.7%) in the post-intervention group (*P* = .014). Prescribing patterns for other alternate antibiotics did not differ significantly based on statistical analyses (Table 4).

**Table 3.** Secondary outcomes

	Pre-intervention CRAB (*N* = 14)	Post-intervention CRAB (*N* = 8)	*P*-value	Pre-intervention *S. maltophilia* (*N* = 75)	Post-intervention *S. maltophilia* (*N* = 75)	*P*-value
All-cause 30-day mortality *n* (%)	2 (14)	1 (12.5)	0.91	18 (24)	31 (41)	0.29
Hospital admission within 90 day *n* (%)	4 (28.5)	3 (37.5)	0.67	17 (22.7)	15 (20)	0.69
Clinical efficacy *n* (%)	11 (78.5)	6 (75)	0.85	52 (69)	60 (80)	0.133
Recurrence of organism within 90 days n (%)	4 (28.5)	3 (37.5)	0.67	22 (29)	24 (32)	0.72
**Antimicrobial AE**						
Acute kidney injury n (%)	2 (14)	0 (0)	0.26	3 (4)	3 (4)	1
Hyperkalemia *n* (%)	--	--	--	2 (2.7)	3 (4)	0.65
Thrombocytopenia *n* (%)	--	--	--	1 (1.3)	2 (2.7)	0.56
Tremors *n* (%)	--	--	--	1 (1.3)	0 (0)	0.32
Rash *n* (%)	--	--	--	1 (1.3)	1 (1.3)	1
GI intolerance *n* (%)	--	--	--	1 (1.3)	1 (1.3)	1
QTc prolongation *n* (%)	--	--	--	1 (1.3)	0 (0)	0.32
Tongue Numbness *n* (%)	1 (7)	0 (0)	0.44	--	--	--
Discontinuation of antimicrobial due to AE *n* (%)	--	--	--	--	--	--

AE, Adverse Effect; GI, Gastrointestinal.

**Table 4.** Antimicrobial agents prescribed

Antibiotic	Pre-intervention CRAB (*N* = 14)	Post-intervention CRAB (*N* = 8)	*P*-value	Pre-intervention *S. maltophilia* (*N* = 75)	Post-intervention *S. maltophilia* (*N* = 75)	*P*-value
Minocycline *n* (%)	5 (35.7)	5 (62.5)	1	1 (1.3)	17 (22.7)	<0.001*
Ampicillin/sulbactam (%)	5 (35.7)	5 (62.5)	1	--	--	--
Colistimethate *n* (%)	3 (14)	0 (0)	0.16	--	--	--
Levofloxacin *n* (%)	4 (28.6)	0 (0)	0.95	49 (65)	26 (34.7)	<0.001*
SXT *n* (%)	2 (14)	2 (25)	1	26 (34.7)	41 (54.7)	0.014*
Amikacin *n* (%)	1 (7)	0 (0)	0.44	--	--	--
Meropenem *n* (%)	2 (14)	2 (14)	1	2 (2.7)	0 (0)	0.16
Cefiderocol *n* (%)	0 (0)	1 (12.5)	0.44	--	--	--
Ceftazidime *n* (%)	--	--	--	8 (10.7)	1 (1.3)	0.016*
Ceftazidime/avibactam + aztreonam *n* (%)	--	--	--	0 (0)	3 (4)	0.80

*P < .05.

SXT, sulfamethoxazole/trimethoprim.

## Discussion

This study evaluated treatment strategies and outcomes for CRAB and *S. maltophilia* before and after implementation of institutional treatment guidance. This study demonstrated a significant change in the way infections caused by CRAB and *S. maltophilia* were treated following the publication of a treatment algorithm on our internal antimicrobial stewardship website and after education to the ID Division. A previous study that evaluated guideline implementation for the treatment of skin and soft tissue infections suggested that implementing an evidence-based guideline may improve prescribing of empiric antimicrobials.^9^

A single-center, retrospective study conducted by Pant et al. assessed the adherence to institutional treatment guidelines for several infection types including pneumonia, urinary tract infections, and skin and soft tissue infections.^10^ Their study showed an adherence rate of 37.9% after publication of treatment guidelines, which highlights the importance for institution-level treatment guidelines.^10^ The rate of adherence in the present study after institutional treatment guidelines was 5/8 (62.5%) for CRAB and 38/75 (50.7%) for *S. maltophilia.* While adherence increased after guideline implementation, there remains significant room for improvement.

As previously mentioned, our treatment guidance document was published on our institution’s antimicrobial stewardship program (ASP) website. In addition to this intervention, repeated educational sessions on local treatment guidelines have also been shown to change empiric antibiotic use.^11^ A study conducted by Smiley et al. improved empiric antimicrobial prescribing for intraamniotic infections via electronic clinical decision support tools and repeated education sessions.^11^ Another study by Zahlanie et al. integrated a computerized order set for outpatient treatment of pediatric bacterial respiratory infections.^12^ In this study, a higher percentage of first-line antibiotic prescribing and shorter durations of treatment were observed after the order set was coupled with educational sessions to providers.^12^ None of the previously mentioned strategies were used in the present study. If electronic clinical decision support tools including order sets and repeated educational sessions are combined with local published treatment guidance, this could possibly further improve targeted therapy for these difficult-to-treat infections.

For notable changes in antimicrobial use, in CRAB there was a decrease in utilization of both polymyxins and aminoglycosides in the post-intervention group. This finding is in concordance with the IDSA document for the treatment of MDR gram-negative infections with sulbactam being a preferred agent for CRAB.^4^ It is also worth mentioning that even though sulbactam-durlobactam was approved during the post-intervention period no patients were treated with this agent. This is likely due to our institution not adding this agent to formulary until February 2024, and providers being unfamiliar with this agent. We also saw more patients treated with SXT and less with levofloxacin for *S. maltophilia.* The decrease in levofloxacin use for *S. maltophilia* is noteworthy, considering that PK/PD data has shown variable activity in isolates with an MIC ≥ 2 and the possibility of isolates developing resistance after levofloxacin exposure.^13,14^

Another significant change that was observed post-intervention was the decrease of ceftazidime monotherapy for patients with *S. maltophilia. S. maltophilia* ceftazidime breakpoints were not removed by CLSI until February 2024 due to intrinsic L1 and L2 β-lactamases expressed by these isolates, which have been shown to hydrolyze ceftazidime.^15,16^ Clinical success reported for ceftazidime monotherapy is as low as 66%.^17^ No differences in clinical outcomes were found in our study, but we anticipate that less ceftazidime and levofloxacin monotherapy may lead to improved outcomes over time. We also acknowledge that even though cefiderocol was included in this treatment algorithm, no patients were treated with this agent for *S. maltophilia.* Cefiderocol has an additional restriction at our institution for approval by antimicrobial stewardship program approval only. It also likely came down to provider or pharmacist preference of using ceftazidime/avibactam + aztreonam over cefiderocol as a metallo-β-lactamase active β-lactam regimen at our institution, and not every patient with an infection caused by *S. maltophilia* being included in our study.

Secondary outcomes for the present study were exploratory and none of them were found to be statistically significant despite an increased number of patients treated with guideline directed antimicrobial therapy for CRAB and *S. maltophilia*. All-cause 30-d mortality and clinical efficacy were similar pre- and post-intervention for CRAB, but slightly higher in the post-intervention group for *S. maltophilia*. A possible explanation of this difference is likely due to the random sampling of patients for the *S. maltophilia* cohorts. Microbiologic recurrence was relatively similar pre- and post-intervention for both organisms which is as expected since both organisms are colonizers. It is noteworthy that no patients in the CRAB post-intervention group experienced an antimicrobial related adverse effect while there were three in the pre-intervention group which were all attributed to either amikacin or colistimethate.

There were several limitations to this study. First, given the retrospective nature of this study, there could have been a selection bias toward certain regimens based on perceived severity of infection or belief that an isolate was more or less likely to represent a pathogen. In addition, we only included a randomly selected sample of patients in the *S. maltophilia* cohort and may have missed treatment or outcome trends only evident with a greater sample size. For secondary outcomes, limitations in interpreting these include small sample size and retrospective methodology. Other limitations of this study include possible confounding factors that we did not include in our analyses or that were not documented in the medical record. Specifically, our microbiology lab stopped reporting ceftazidime susceptibilities for *S. maltophilia* isolates in March 2024 after CLSI removed this breakpoint. Another possible confounding factor is the update of minocycline breakpoints which were lowered in 2023 for *S. maltophilia* from ≤4 µg/mL to ≤1 µg/mL.^15^ This update was based on data showing doses of minocycline 200 mg q12h have a >90% probability of achieving PK/PD targets in neutropenic models for organisms with MICs of 1 of µg/mL.^18^ Both interventions could have resulted in reduced ceftazidime use and higher doses of minocycline for *S. maltophilia.*

In summary, after implementing a local treatment guideline based on IDSA guidance for CRAB and *S. maltophilia* on our institution’s ASP website, we saw an increase in guideline directed antimicrobial therapy post-intervention for both of these organisms. These findings highlight an opportunity for ASP interventions to improve antibiotic selection according to published guidelines and local susceptibility patterns. Additional studies are needed to define the impact of this treatment guidance on clinical outcomes. Future work should also include the intervention of repeated educational sessions combined with electronic order sets in addition to institutional treatment algorithms to further improve treatment guidance adherence.

## Supporting information

10.1017/ash.2025.10244.sm001 Vathy et al. supplementary material 1 Vathy et al. supplementary material

10.1017/ash.2025.10244.sm002 Vathy et al. supplementary material 2 Vathy et al. supplementary material

## Data Availability

The authors confirm that the data supporting the findings of this study are available within this manuscript and its supplementary materials.

## Supplementary material

The supplementary material for this article can be found at https://doi.org/10.1017/ash.2025.10244.
