# Supplementary material for: Impact of institutional treatment guidance on the management and outcomes of Stenotrophomonas maltophilia and carbapenem-resistant Acinetobacter baumannii infections
Source: Antimicrob Steward Healthc Epidemiol. 2025 Dec 10;5(1):e330. doi: 10.1017/ash.2025.10244 (PMC12722545; doi:10.1017/ash.2025.10244)

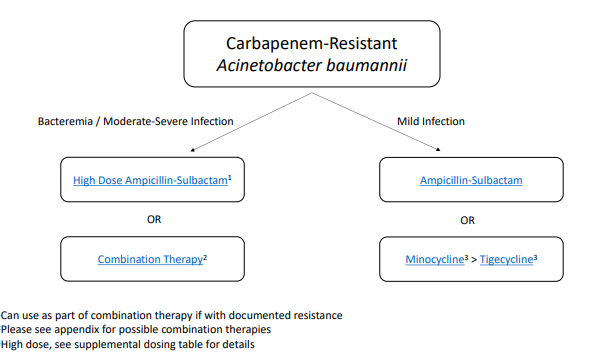


Treatment algorithm for Carbapenem-Resistant *Acinetobacter baumannii*


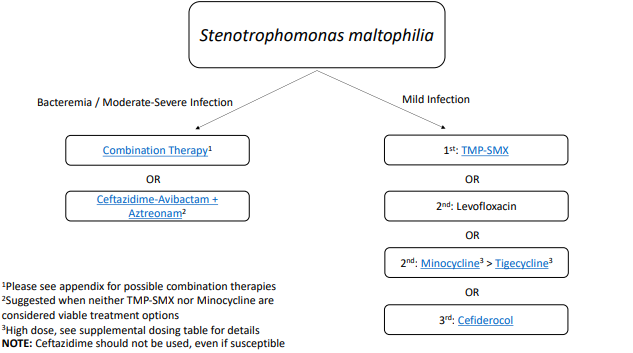
Treatment algorithm for *Stenotrophomonas maltophilia*


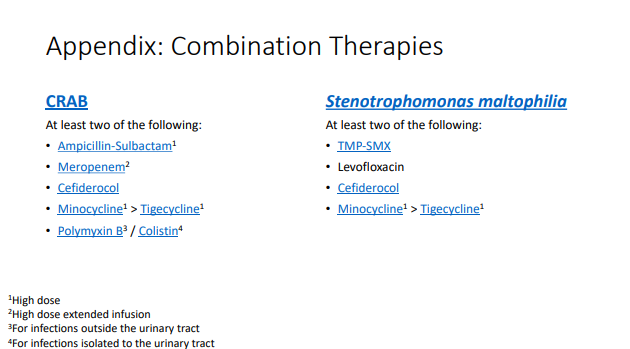
Recommended combination therapies


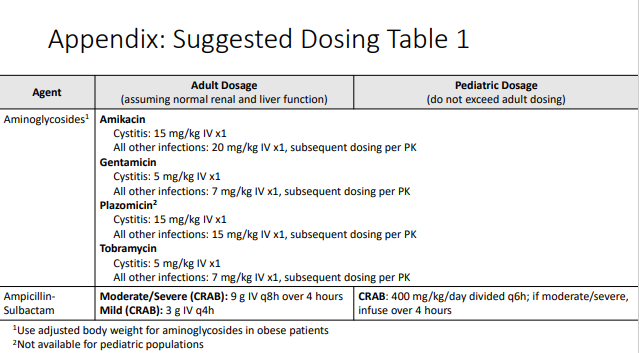


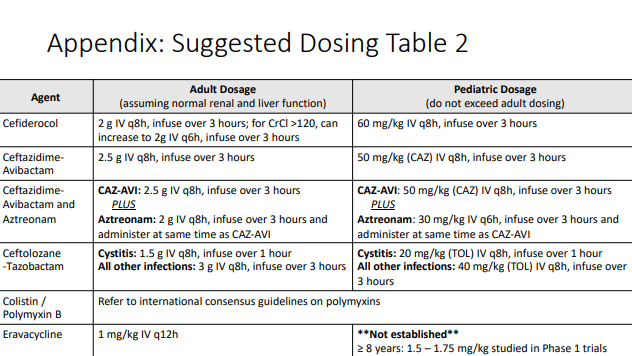


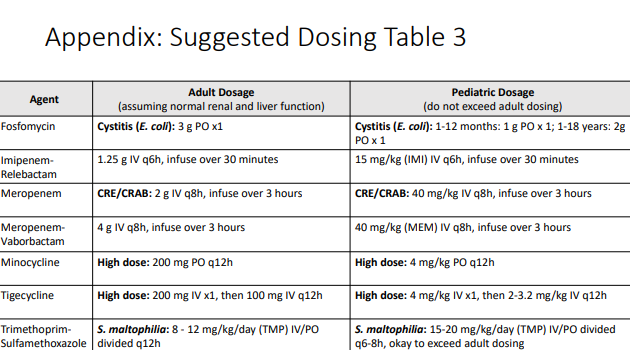

Supplement: Vathy et al. supplementary material 1 — Vathy et al. supplementary material [file S2732494X25102441sup001.docx]
