# Supplementary material for: Impact of institutional treatment guidance on the management and outcomes of Stenotrophomonas maltophilia and carbapenem-resistant Acinetobacter baumannii infections
Source: Antimicrob Steward Healthc Epidemiol. 2025 Dec 10;5(1):e330. doi: 10.1017/ash.2025.10244 (PMC12722545; doi:10.1017/ash.2025.10244)
Supplement: Vathy et al. supplementary material 2 — Vathy et al. supplementary material [file S2732494X25102441sup002.docx]

**Appendix Table: Antibiotic Dosages**

| **Antibiotic** | **Pre-** **Intervention** **CRAB**  **(N = 14)** | **Post-Intervention CRAB**  **(N = 8)** | **Pre-Intervention**  ***S. maltophilia***  **(N = 75)** | **Post-Intervention *S. maltophilia***  **(N = 75)** |
| --- | --- | --- | --- | --- |
| **Minocycline n (%)** | | | | |
| 100 mg q12h | 5 (36) | 0 (0) | 1 (1.3) | 7 (9.3) |
| 200 mg q12h | 0 (0) | 5 (62.5) | 0 (0) | 10 (13.3) |
| **Ampicillin/sulbactam n (%)** | | | | |
| 1.5g q12h | 1 (7) | 0 (0) | -- | -- |
| 3g q8h | 1 (7) | 0 (0) | -- | -- |
| 3g q6h | 1 (7) | 1 (12.5) | -- | -- |
| 3g 4h | 2 (14) | 0 (0) | -- | -- |
| 9g q12h | 0 (0) | 1 (12.5) | -- | -- |
| 9g q8h | 0 (0) | 3 (37.5) | -- | -- |
| **Colistimethate n (%)** | | | | |
| 1.4 mg/kg q8h | 1 (7) | 0 (0) | -- | -- |
| 1.3 mg/kg q12h | 1 (7) |  | -- | -- |
| 1.7 mg/kg q12h | 1 (7) | 0 (0) | -- | -- |
| **Levofloxacin n (%)** | | | | |
| 500 mg q48h | 1 (7) | 0 (0) | 6 (8) | 4 (5.3) |
| 750 mg q48h | -- | -- | 7 (9.3) | 4 (5.3) |
| 500 mg q24h | -- | -- | 5 (6.7) | 3 (4) |
| 750 mg q24h | 3 (21) | 0 (0) | 31 (41) | 15 (20) |
| **SXT (TMP component) n (%)** | | | | |
| < 5 mg/kg/d | -- | -- | 6 (8) | 2 (2.7) |
| 5-8 mg/kg/d | 1 (7) | 1 (12.5) | 4 (5.3) | 13 (17.3) |
| 8.1-12 mg/kg/d | 0 (0) | 1 (12.5) | 10 (13.3) | 23 (31) |
| > 12.1 mg/kg/d | 1 (7) | 0 (0) | 6 (8) | 3 (4) |
| **Amikacin n (%)** | | | | |
| 7 mg/kg (spot dosing) | 1 (7) | 0 (0) | -- | -- |
| **Meropenem n (%)** | | | | |
| 1g q8H | 1 (7) | 1 (12.5) | 1 (1.3) | 0 (0) |
| 2g q8H | 1 (7) | 1 (12.5) | 1 (1.3) | -- |
| **Cefiderocol n (%)** | | | | |
| 2g q8h | 0 (0) | 1 (12.5) | -- | -- |
| **Ceftazidime n (%)** | | | | |
| 1g q24h | -- | -- | 2 (2.7) | 0 (0) |
| 2g q24h | -- | -- | 1 (1.3) | 1 (1.3) |
| 2g q8h | -- | -- | 5 (6.7) | 0 (0) |
| **Aztreonam n (%)** | | | | |
| 1g q12h | -- | -- | 0 (0) | 1 (1.3) |
| 2g q8h | -- | -- | 0 (0) | 2 (2.7) |
| **Ceftazidime/avibactam n (%)** | | | | |
| 0.94 g q8h | -- | -- | 0 (0) | 1 (1.3) |
| 1.25 gm q8h | -- | -- | 0 (0) | 1 (1.3) |
| 2.5 gm q8h | -- | -- | 0 (0) | 1 (1.3) |

SXT = Sulfamethoxazole/Trimethoprim
